# Towards precision medicine; a new biomedical cosmology

**DOI:** 10.1007/s11019-018-9828-z

**Published:** 2018-02-10

**Authors:** M. W. Vegter

**Affiliations:** Faculty of Science, Institute for Science in Society, Radboud University Nijmegen, P.O. Box 9010, 6500 GL Nijmegen, The Netherlands

**Keywords:** Precision medicine, Medical cosmology, Foucault, Big data, Participatory medicine, ’All-of-Us’ research program

## Abstract

Precision Medicine has become a common label for data-intensive and patient-driven biomedical research. Its intended future is reflected in endeavours such as the Precision Medicine Initiative in the USA. This article addresses the question whether it is possible to discern a new ‘medical cosmology’ in Precision Medicine, a concept that was developed by Nicholas Jewson to describe comprehensive transformations involving various dimensions of biomedical knowledge and practice, such as vocabularies, the roles of patients and physicians and the conceptualisation of disease. Subsequently, I will elaborate my assessment of the features of Precision Medicine with the help of Michel Foucault, by exploring how precision medicine involves a transformation along three axes: the axis of biomedical *knowledge*, of biomedical *power* and of the patient as a *self*. Patients are encouraged to become the managers of their own health status, while the medical domain is reframed as a data-sharing community, characterised by changing power relationships between providers and patients, producers and consumers. While the emerging Precision Medicine cosmology may surpass existing knowledge frameworks; it obscures previous traditions and reduces research-subjects to mere data. This in turn, means that the individual is both subjected to the neoliberal demand to share personal information, and at the same time has acquired the positive ‘right’ to become a member of the data-sharing community. The subject has to constantly negotiate the meaning of his or her data, which can either enable self-expression, or function as a commanding Superego.

## Introduction

Since October 2016, The Precision Medicine Initiative has been re-labelled as the ‘All of Us’ research program (AoU). The collection of data from more than a million Americans, coming from medical files and biological samples, but also from smartphones and other personal devices, allegedly offers researchers new insights in the onset of disease. The AoU program is part of a bigger trend where biomedical research increases its technological capacity through the use of Big Data, while at the same time incentives are developed inviting citizens to become part of this ‘sharing’ community. Both the focus on big data and the emphasis on ‘participatory’ medicine (framing participants as research partners, providing mobile health data) reflect new features currently arising in the biomedical domain.

In this paper I will address the question whether this constellation of individualised healthcare and big data within precision medicine (exemplified by the AoU program) can be regarded as a new biomedical paradigm, or even a new cosmology, a concept introduced by Nicholas Jewson in 1976 and explained in more detail below. To address this overall question, I will first of all analyse the AoU design as an architecture reflecting the promising future of ‘Precision Medicine’. Precision Medicine is the common label currently in use for biomedical research that focuses on data streams, a significant amount of which is freely shared between individuals. But the terminology is still evolving, so that besides ‘precision medicine’ also ‘personalized medicine’, ‘pharmacogenomics’ and ‘P4 medicine’ (preventative, predictive, participatory, and personalized) capture various aspects of the current biomedical research climate. These conceptualisations all seem to move in the same direction, thus setting the stage for what will be referred to here as precision medicine. Various scholars have already contributed to explaining the context and meaning of Precision Medicine and I will notably build on the work of (amongst others) Boenink and Vogt (Boenink 2009; Vogt et al. 2016a, b). In this paper I will argue, following Boenink, that ‘precision’ captures the ambitions and hopes that come with this new data-driven and participatory turn, although ‘stratified’ (rather than ‘individualized’) medicine would convey a more realistic outcome. In other words, ‘precision’ rather than ‘individualisation’ seems the overarching concept.

Secondly, Precision Medicine will be assessed against the backdrop of a longer history of biomedical constellations, as analysed by Michel Foucault and others. Building on these previous endeavours, I will try to understand the present by assessing it against the backdrop of a broader temporal horizon. Therefore, I will present a concise archaeology of previous conceptual frameworks that have emerged since the dawn of modern medicine, building on the work of Jewson (1976) and his followers (Armstrong 1995; Nettleton 2004) as well as on Foucault. Jewson refers to these consecutive conceptual constellations or frameworks as ‘cosmologies’ and describes them as worlds of practices and discourses, each with a particular profile of its own. In his seminal publication, Jewson distinguished three cosmologies, namely *Bedside Medicine, Hospital Medicine* and *Laboratory Medicine*. Additional cosmologies, namely *Surveillance Medicine* and *E-scaped Medicine*, were later added by Armstrong and Nettleton respectively. These earlier cosmological profiles will allow me to determine the basic features of ‘precision medicine’ as the most recent cosmology. Notably, they allow me to assess the techno-scientific ideals reflected by the AoU program, as key features of precision medicine as such.

I will draw on the work of Foucault, notably by emphasising three dimensions of the precision medicine cosmology; ‘Knowledge’, ‘power’ and ‘self’ (Foucault 1984; cf.; Zwart 2005, 2016a). The first dimension (the knowledge axis) focusses on the epistemological developments and allows me to discuss some potential benefits and weaknesses of big data.(Leonelli 2014). As for the power dimension, I will analyse and assess how power is redistributed when bioinformatics opens up the domain to various commercial actors (Swan 2009; Zwart 2016b). As to the third axis, the technologies of Self, I will address the focus on wellness and the language of empowerment that accompanies participatory medicine. I will conclude that the debate surrounding precision medicine should address the tensions entailed in collecting, processing and interpreting large-scale (‘big’) health data.

## A new window for viewing health

The Precision Medicine Initiative (PMI) was launched by the Obama Administration in 2015 together with a publication of the Precision Medicine Cohort Program by Francis Collins and Harold Varmus in the *New England Journal of Medicine* (Collins and Varmus 2015; Hampel et al. 2017). Subsequently, what started as the PMI was translated into the All-of-Us cohort study (AoU). In order to study the AoU design I have analysed the official documents on the NIH government website, such as ‘Precision Medicine Initiative (PMI) Working Group Report to the Advisory Committee to the Director, NIH’ (PMI Working Group 2015). Additionally, I have looked into a number of perspective papers (Ashley 2016; Collins and Varmus 2015; Hamburg and Collins 2010; Mirnezami et al. 2014; Peterson et al. 2013). I consider the AoU as the actualisation (concrete and physical) of the very ideals that inspired precision medicine discourse from the outset. Similar initiatives such as ‘The precision medicine initiative for Alzheimer’s disease’, or the ‘100 k Wellness Project’ have been developed (Hampel et al. 2017; Hood and Price 2014; Vogt et al. 2016a, b).

Similar initiatives are also mentioned in the 2015 PMI-working group publication such as the UK Biobank, The china Kadoorie Biobank and the Estonian endeavor to link a population biobank with national health registries (Chen et al. 2011; Leitsalu et al. 2015; PMI Working Group 2015, p. 19; Sudlow et al. 2015).

The AoU program focuses on a shift from traditional research and drug development towards developing an overarching structure that includes big data science, systems biology, genomic sequencing, blood-based biomarkers, integrated disease modelling and P4 medicine (Hampel et al. 2017; NIH 2016). The AoU aims to employ the wide-spread use of mobile devices and social media for encouraging healthy behaviours (NIH Gov. link 2016, p. 16). New approaches to patient participation and empowerment are forged via partnerships with a plethora of patient groups. Since October 2016 the cohort is called ‘All-of-Us’. It began enrolling participants in 2017 and is expected to reach one million volunteers within three to four years. Participating individuals play a decisive and active role. Sharing data and linking these data to health records is expected to lead to “the right drug, at the right dose to the right patient” (Collins and Varmus 2015). The ‘precision’ label (instead of the ‘personalised medicine’ label) is used to prevent the expectation that medication will be synthesized personally for individual patients (Ashley 2015; National Academy of Sciences (NAS) 2011). Rather, precision medicine is about creating subcategories of disease, thus furthering stratification (Boenink 2016). But this is seen as a major step forward. For although statistical analysis provides evidence for the safety and effectiveness of various FDA-approved drugs, in practice only limited numbers of patients respond positively to evidence-based treatments. Stratification into subcategories could improve these outcomes and, as a result, improve treatment while reducing the risks of side-effects.

Initially, the term ‘personalised medicine’ was used for efforts to produce a more individualised understanding of health and disease on the basis of genetic profiles, shifting from symptom-based to genome-based approaches (National Academy of Sciences (NAS) 2011; Prainsack 2015). As indicated, the recently launched ‘All of Us’ program prefers the ‘precision’ label, but likewise focusses on opportunities for developing quantitative, individualised estimates of risk for a range of diseases. Several scientific opportunities are presented by the NIH for the ‘all of Us’ cohort, (National Institutes of Health, n.d.) (PMI Working Group 2015, pp. 15–18). It is expected to:

- Develop ways to measure risk for a range of diseases based on environmental exposures, genetic factors and interactions between the two.
- Identify the causes of individual differences in response to commonly used drugs (pharmacogenomics);
- Discover biological markers that signal increased or decreased risk of developing common diseases;
- Use mobile health (mHealth) technologies to correlate activity, physiological measures and environmental exposures with health outcomes;
- Develop new disease classifications and relationships;
- Empower study participants with data and information to improve their own health;
- Create a platform to enable trials of targeted therapies.

According to the official White House document ‘*Precision Medicine Initiative: Data Security Policy Principles and Framework’* the types of data used for these PMI activities could include, but are not limited to, clinical and insurance claims data, surveys and demographic data, genomic and other biosample-derived data, and mobile, implantable, or other equipment or device data, all of which may be stored and processed electronically (The White House 2016).

These PMI data include information about the participant’s medical history and lifestyle, but participants may also be asked to provide physical measurements (blood pressure, height and weight, etc.) at a local enrollment center, or donate blood and urine samples (NIH 2018a).

In other words, precision medicine combines population research with various forms of molecular mapping, giving rise to big data. Thus, it is expected to involve various transformations on the level of *vocabularies and labels* (taxonomy), but also on the level of *roles for patients and physicians* (increased emphasis on patient responsibility in terms of self-monitoring and self-management), eventually even changing the way in which medicine is organized.

The AoU Initiative was launched in the midst developments and discourses on personalized medicine, pharmacogenomics, P4 medicine, systems medicine, and developments in the field of iPOP for example^1^ (Chen et al. 2012; Leroy et al. 2012; PMI Working Group 2015). It may therefore be regarded as exemplary, as it demonstrates how thinking about genomics in the aftermath of the Human Genome Project (HGP) is translated into a concrete initiative. The ‘All of Us’ captures the contemporary conviction that research should function at the intersection of lifestyle, environment and biological make-up. Recent extensions of the P’s in P4 medicine by adding a fifth P (‘*always taking into account a Population perspective*’) or even a sixth P (the psycho-cognitive P) is indicative of the fact that the changes are still ongoing; it tells us that something is happening (Anaya et al. 2016; Kondylakis et al. 2014). The suggestion that we are in an ‘*epoch of shared decision making’* should be critically analyzed however in the context of ‘participatory’ medicine (Kondylakis et al. 2014).

Boenink offers an important view on recent developments concerning Precision Medicine (Boenink 2016). According to Boenink we have taken a turn in the direction of molecular medicine that entails a shift towards a focus on ‘biomarkers’ rather than genetics and genomics. In her chapter ‘Disease in the Era of Genomic and Molecular Medicine’ she considers some of the main features of health and disease, focusing on the underlying technology. While the HGP encouraged ‘geneticization’ (Lippman 1991) because the underlying view of genetics was monocausal and deterministic in its understanding of the relationship between genes and disease, molecular medicine moved away from such a deterministic view; from genes to genome to multiple -omics. As a result of what Zwart called a narcissistic offence—the realization that the human genome is not as exceptional as was initially expected—biomedicine started to look for more complex models of disease (Zwart 2007). Boenink calls this approach a ‘cascade model of disease’, which is made possible by the development of molecular biology and nanotechnology; because research can now ‘zoom-in and quantify’ what is happening, and secondly through the development of biobanks, software and algorithms that serve as a necessary background, providing the context for this quantification. Here also, biobanks and bioinformatics usually require computation, using clear-cut categories to describe disease and health. These developments, pointed out by boenink, are mentioned in the 2015 PMI-working group report as well, stating that the information technology revolution has provided remarkable reductions in the cost of data storage. Meanwhile, the raw cost of DNA-sequencing has been reduces nearly 10-million-fold from the time the sequencing phase of the Human Genome Project began in 1998 (PMI Working Group 2015, p. 8). Both the zoom-in and the zoom-out strategies mediate a ‘quantitative view of disease’, where disease has become a deviation of a population mean. The goal of biomedical research has therefore become to explore normal functioning.

Boenink concludes by pointing out two shifts, in line with Leroy Hood’s work (Leroy Hood and Flores 2012), although Boenink’s assessment of them is more critical than Hood’s. One is that there is no real boundary between health and disease, the other is that a cascade model enables high precision claims concerning prediction and prevention.

Hood offers a personal and promising view on prediction within systems medicine and the emergence of P4 medicine (Leroy Hood and Flores 2012). Hood envisions to ‘*make blood a diagnostics window for viewing health and disease for the individual*’. Another objective is to ‘*Generate metrics for assessing wellness*.’ This view is in accordance with what in the AoU is seen as scientific opportunities such as discovering biomarkers that identify individuals with an increased risk of developing common diseases, but also the opportunity to develop new disease classifications and relationships and promote healthy behavior (p. 15). In response to these developments Vogt et al. argue that health and illness are seen as something concrete, objective and already *there* (Vogt et al. 2016a, b) and they criticize Hood and Flores’ view that disease and wellness can be seen as a continuum of network states (unique in time and space for each individual human being), captured in biometrics. According to these authors the concept of complexity is reduced to a technoscientific version of holism; although the process of life is defined as complex, it is still defined in terms of quantifiability, predictability and actionability; and therefore appears controllable. Moreover, the authors warn that; *‘it is a fallacy to assume that providing a quantitative correlate to a construct that is already normatively defined automatically makes it objective, purely scientific or non-normative*.’ (Vogt et al. 2016a, b, p. 413). Thus, they urge us to be more suspicious concerning the technological backdrop of these numbers.

In summary, precision medicine is the general idea that the more health data we gather, the more we can quantify, the more we can control. In consequence, the boundary between health and disease becomes obscured. It is suggested that we can intervene at various stages in a variety of molecular cascades. On the normative level, precision medicine entails the idea that adequate health management will allow individuals to increase their chances of remaining within the healthy strata of the population.

## Theoretical framework: medical cosmology

Before analysing Precision Medicine as a medical cosmology, I will first of all describe the methodological and conceptual background of the cosmology approach. In 1976 Nicholas Jewson described a series of transformations in how disease and illness had been conceptualized during the previous two centuries. Notably, Jewson pointed out that medical knowledge has gradually become increasingly abstract and distant, due to changes in the “mode of production” of medical knowledge. With each successive step, the distance between caregiver and patient, and between lab bench and bedside, had increased. Jewson described the medical world as a cosmology, a constellation of practices, vocabularies, institutional networks, etc. and the general trend which he discerned in modern history was a gradual “*disappearance of the sick man from medical cosmology*” (Jewson 1976). Rather than in specific practices or specific semantical issues, Jewson was interested in outlining the cosmology *as a whole*, the emergence of new constellations within the medical domain, involving new physician-patients interactions as well as new vocabularies and taxonomies.^2^

Cosmologies can be seen as a set of axioms and assumption which guide the interests of medical investigators. Therefore it is a way of seeing, but also of not seeing. A medical cosmology should not be regarded as a static normative framework, but rather as an evolving set of possibilities and impossibilities (inclusions and exclusions). It was Jewson’s specific interest to show that medical knowledge entails active knowing and is therefore a mode of social interaction. The medical cosmology is the medium within and through which perceptions of self and others are expressed, legitimized and institutionalized. In summary, medical cosmologies are not only statements about the world, but enactments of ways of relating to others in this world.

The profile of a cosmology is dependent upon a distinction that is made from the very beginning; a cosmology can be structured around either persons or objects. The constellation of meaning assigned to medical events depends on this. According to Jewson, person-oriented cosmologies provide a wide range of alternatives for the expression and realization of meanings, and he speaks of letting each individual develop his or her own particular perception of a body-self. Object-oriented cosmologies function differently. In such a cosmology, persons relate to one another in terms of the social categories to which they belong, so that they are treated as if they were things or objects. Jewson states *‘The study of life is replaced by the study of organic matter.’* Modern medicine, he argues, is focused upon the recurring, objective, quantitative characteristics of categories of the sick rather than upon the unique, subjective, qualitative difference between individuals. By knowing how medical knowledge is ‘made’, we can learn what these evolving relations look like.

The term cosmology indicates that Jewson’s objective was not to write a history of medicine, but rather to develop a typology, a temporal sequence of types or styles of medical practice, comparable to, for instance, to the paradigm concept of Kuhn or the episteme-concept of Foucault. But whereas Kuhn’s paradigm concept focusses on science and scientific worldviews, Jewson’s cosmologies involve practical, clinical and ethical aspects as well (Greaves 2002; Nicolson 2009). Jewson’s approach is comparable to Foucault’s archaeology, as developed in *The Birth of the Clinic* (Foucault 1963) and other writings. It is not a purely descriptive endeavour, but entails a critical analysis, a critical diagnostics, allowing us to assess the epistemological and ethical strengths and weaknesses of particular cosmologies. And indeed, in his paper on surveillance medicine, discussed below, Armstrong (1995) not only introduced a fourth cosmology, but also explicitly connected Jewson’s analysis of medical *cosmologies* with Foucault’s analysis of medical *topologies*.

Building on this body of work by Jewson and others, the question that will be addressed in this paper is whether precision medicine can be represented as a cosmology of its own and, if so, how it differs from previous cosmologies; what are the defining features of Precision Medicine, represented as a cosmology? New technologies prepare the ground for the emergence of new medical cosmologies, but they never fully replace the previous ones. Rather, each successive cosmology absorbs specific features of previous cosmologies, embedding them into new constellations. Jewson speaks therefore of an ‘eclipse’ of bedside medicine by hospital medicine and, subsequently, of hospital medicine by laboratory medicine. In each medical cosmology, biomedical researchers and research subjects are positioned in a certain way. Yet, new ways of ‘knowing’ reshape these relationships and the positions these actors have, relative to the institutions to which they belong. We can speak of a new cosmology when new technologies and new forms of biomedical knowledge give rise to a reorganisation of the elements or the objects within the existing cosmology. As Pickstone phrases it: cosmologies should not be seen as successive types of medicine, but rather as inter-penetrating types, where novel forms of medicine co-exist with the old in contested cumulations (Pickstone 2009; Tutton 2012).

I will now briefly describe the overall profile and key components of these five cosmologies before turning attention to Precision Medicine. Table 1 offers a concise overview of these five profiles. According to Jewson, *Bedside Medicine* emerged around 1770. In this cosmology, the patient was regarded as a totality, a person. Patient and physician developed an interpersonal relationship and medical care was provided in response to the specific needs and wishes of the individual patient (provided he or she was able to pay for this type of care).

**Table 1:** Medical cosmologies

	Occupational role of physician	Perception of the “sick man” (i.e. patient)	Task of biomedical investigator	Conceptualisation of illness
Bedside	Practitioner	Person	Prognosis and therapy	Overall psycho-somatic disturbance
Hospital	Clinician	Case	Diagnosis and classification	Organic lesion
Laboratory	Scientist	Cell complex	Analysis and explanation	Biochemical process
Surveillance	Epidemiologist	Risk assemblage	Conversion of epidemological risk toclinical risk	Latent deviation from norm
E-scaped	Information scientist	Expert patient health seeker	Assessment and communication of risk evidence	Communication breakdown

*Hospital Medicine* emerged in the early nineteenth century, when medicine attempted to develop a more formal knowledge structure in order to guide professional practice. Hospital medicine focused on pathology and accurate diagnostics, rather than on care or therapy. The sick person became a “collections of organs”, exposed to a scientific gaze, an analysis which to some extent concurs with Foucault’s description of the “birth of the clinic” (Foucault 1963). And indeed, Foucault likewise considers the year 1800 as an important turning-point.

During the final decades of the nineteenth century, however, this focus on pathology and diagnostics was significantly strengthened by laboratory research, and this resulted in the emergence of a third cosmology, namely *Laboratory Medicine*, which again entailed a drastic conceptual and practical reframing of health and disease. From now on, with the help of X-rays and blood samples for instance, disease was located in microscopic events, brought to the fore via biomedical technology (Rosenberg 2007, pp. 19–20). Illness was transferred as it were from the bedside into the extra-corporeal laboratory realm.

Building on Jewson’s seminal publication, Armstrong (1995) subsequently presents a fourth cosmology, namely *surveillance medicine*, focussed on monitoring the health status of populations (rather than individual patients) and the distribution of disease within populations. According to Armstrong, illness is now redefined in terms of individual deviations from statistical norms and thus involves ‘*a problematisation of the normal*’. Illness becomes a statistical phenomenon, describable as the relative position of an individual within a population; illness is captured in terms of ‘risk’ and ‘lifestyle’. This is not only a theoretical reorganisation, for it also affects the organization of medicine, resulting in the development of new types of interventions, new institutions and a new relation to the self. As was already indicated, Armstrong combines the cosmology concept developed by Jewson with the topological archaeology of Foucault, emphasising that surveillance medicine entails a very specific “spatialisation” of disease, a “remapping” of illness, reflecting a shift of the medical gaze into extra-corporal space, a shift of focus from observing hospitalised patients to monitoring interactions between individuals in seemingly healthy populations.

In addition, a more recent cosmology has been put forward by Nettleton (2004), namely “E-scaped medicine” (2003), based on the rise of the internet as a global archive of biomedical information and as a means for communication (i.e. web 1.0, in combination with email and use of webfora). Her analysis is inspired by de Mul’s work on ‘the informatization of the worldview’ (Mul 1999). Due to the internet and other infrastructures, she argues, medical information becomes accessible to broad audiences in novel ways. Medical knowledge spreads to multiple virtual locations. Lay people (patients and healthy citizens) can access the medical domain from anywhere, so that medical information becomes de-institutionalized. For Nettleton, the informational turn explains a turn towards Evidence-Based Medicine as a central organizing concept (in opposition to the ‘art of medicine’). Besides changing medical practice, this also prepares the ground for problem-based learning in university curriculums, and explains why clinical decision-making increasingly relies on communication and information interchange (cf. Nicolson 2009). A focus on analysability, programmability and controllability is central to medical knowledge within this cosmology rather than ‘mechanical medicine’ (Lupton 2012; Nettleton and Burrows 2003).

These cosmologies can be summarised in shorthand in the form of the following diagram, a concise version of the diagram developed by Nettleton (2004), which was an extension of Jewson’s original diagram^3^ (1976, p. 228). Thus, this table summarises a whole body of literature.

## Precision medicine as a biomedical cosmology of the present

In this section I will address Precision Medicine as an emerging medical cosmology. What are the key features of precision medicine? As indicated by the headings shown in Table 1, a new medical cosmology entails a new conceptualization of disease that not only changes the role and task of physicians and biomedical investigators, but also entails a new perception of the target of biomedical interventions, referred to by Jewson as “the sick man”.^4^ In terms of the cosmology concept, Precision Medicine emerges as a new constellation at the intersection of laboratory medicine, surveillance medicine and e-scaped medicine, each of them contributing some specific conceptual features that are reassembled under the new heading of precision medicine. It combines a focus on molecular medicine with the use of large-scale population data and a decidedly informational orientation.

The following quote for instance indicates the expectation that, building on previous forms of knowledge (i.e. cosmologies), the AoU will develop a new disease classification, one that is sensitive to molecular characterization and the availability of large data sets;

Current classifications of disease typically group symptoms, signs, and laboratory results into a discrete diagnostic category. Underlying these structures is a disease nomenclature anchored in centuries of observation prior to the current era of molecular characterization. A large and complex set of data points from one million or more participants, including comprehensive clinical records, a broad range of laboratory and molecular investigations, and clinical diagnoses and health outcomes, provides the opportunity to discover unexpected connections within the data as well as new subtypes of disease. (PMI Working Group 2015, 17)

At the same time it shows that these former cosmologies are ‘eclipsed’ by precision medicine, notably by the belief in big data science. How the PMI envisions the expanding wings of Big Data is apparent in the following quote;

The PMI cohort is being launched at a time of explosive growth in the number, size, and complexity of potentially relevant data resources. The “big data” of human biology, such as full genomes and high resolution digital images, may be combined with other novel forms of equally large or larger data, such as weather patterns, environmental monitoring, and streaming physiologic sensor data from study participants. P. 67

Through the combination of data as such, these former cosmologies converge [or cumulate (Pickstone 2009)] into Precision Medicine as a new constellation, a new combination of continuity and discontinuity, of existing and innovative features. As indicated earlier, Precision Medicine and its focus on biomarkers foresee opportunities for intervention and prediction, and ultimately self-management through mobile health devices. This will allegedly enable a whole new set of unexpected assosciations, as is reflected in the following quote;

The PMI cohort will provide a broadly useful resource for rigorously validating and quantifying the contributions of genetic and environmental risk factors, as well as their interactions with one another, in a large, diverse population. This will certainly include risk factors that have been proposed from smaller studies, but the comprehensiveness of the PMI cohort dataset will also allow for data scientists to identify new and unexpected associations. As it grows in breadth and depth, the PMI cohort will allow for these estimates on uncommon as well as common diseases. p. 15.

These claims and promises allow a reconceptualization of the categories of Table 1, expressed in Table 2. The emerging cosmology implies a new role for physicians and patients, for both are now involved in the collection and processing of enormous amounts of data. While doctors have to interpret health data and consult their patients, patients are actively taking up mobile health and self-monitoring practices in order to improve their health and participate in the research process (PMI Working Group 2015, pp. 62, 63) Although ultimately it is the professional’s job to suggest biomarker-based behavioural change (in terms of life-style, diet, medication etc.), individuals themselves are to decide what technology to purchase and which data to process, ultimately rephrasing the subject in terms of digital consumption, staging them as digital consumers. In the AoU this is captured by a vision that participants become ‘partners’ in managing their health.

**Table 2:** Medical cosmologies; precision medicine

Cosmology	Occupational role of physician	Perception of ‘sick man’ (i.e. patient)	Task of biomedical investigator	Conceptualization of Illness
Bedside	Practitioner	Person	Prognosis and therapy	Overall psycho-somatic disturbance
Hospital	Clinician	Case	Diagnosis and classification	Organic lesion
Laboratory	Scientist	Cell Complex	Analysis and explanation	Biochemical process
Surveillance	Epidemiologist	Risk assemblage	Convert epidemiological risk to clinical risk	Latent deviation from norm
E-scaped	Information scientist	Expert Patient Health Seekers	Assessment and communication of risks; and assessment of research evidence	Communications breakdown, interaction of systems
Precision	Data-consultant	Digital consumer	Biomarker based health promotion	Ill-managed molecular cascades

A goal of the PMI cohort is to empower individuals to understand potential opportunities to manage their health offered through genomic sequencing, aggregation of longitudinal health information, and sharing of data with researchers, under a cooperative model of partnership and trust. (PMI Working Group 2015, 40).

This goal (to empower individuals as active consumers) is even more explicitly expressed in the AoU’s objective to use mobile health technology and wearables. One example is the Fitbit pilot that was announced by Director of the AoU program Eric Dishman, who actively seeks an audience within the AoU ‘community’ with the help of YouTube videos and explains his interest to use data from the devices that the participants are already using. (Dishman 2017). The pilot involves an interest in how to pull fitbit-data; Fitbit is a company famous for its activity trackers based on wearable technology that measure data such as number of steps, heartrate, sleeppatterns etc. The information is individual-based, but in order to assess the meaning of this individualised information, it has to be connected with large-scale data repositories. In other words, although precision medicine claims to bring the individual body back into view, the patient’s body actually emerges against the backdrop of data derived from millions of other individuals. The biomedical gaze reverts from the population back to the individual body, but this body now emerges as a database, a valuable and exploitable resource of information. Jewson’s “sick man” has been replaced by consumers of digital information, as was already articulated by Barack Obama, for instance, when he claimed that each household should be able to access their health data (Obama 2015). Table 2 contains an updated version of Table 1, adding a row for Precision Medicine regarded as a medical cosmology.

## Foucault

In Foucault’s oeuvre, three dimensions or axes of inquiry can be distinguished, namely *knowledge, power* and the *Self* (Foucault 1984; Zwart 2005, 2016a). These axes are exemplified by key publications such as, ‘Words and things’ (Foucault 1966), ‘Discipline and Punish’ (Foucault 1975) and ‘The care of the self’ (Foucault 1984) respectively. Zwart (2005, 2016a) has argued that these axes can be used as windows into contemporary technoscience as a new epistemic formation, allowing us to ask three types of questions, namely questions concerning (a) new practices of knowledge, (b) new practices of power and (c) new practices of the Self enabled by contemporary technoscience (Zwart 2005). In this section, I will use these three axes (these three sets of questions) to extend my cosmological analysis of precision medicine.

The relevance of Foucault’s work for cosmology analysis was already recognised by others. In his work on surveillance medicine, Armstrong already pointed to the affinities between the approaches of Jewson and Foucault. Armstrong showed that a focus on risk and lifestyles entailed a reconfiguration of symptoms, signs and illnesses, opening up new space of future understandings of health and disease. Medicine is increasingly enacted in an extracorporeal space, represented by the notion of lifestyle. This gives rise to a new spatialiation; a remapping of illness outside the organic confines of the body. This claim is grounded in Foucault’s observation that a new “epistemic formation” or “episteme” will involve new organisations of space and time, new forms of spatialisation and temporalisation. In the context of precision medicine, key terms such as risk and lifestyle are redefined and absorbed into a new cosmology. Notably, they are defined in terms of massive data streams and fine-grained, highly detailed molecular information.

Thus, in order to deepen our understanding of this particular cosmology, three types of questions must be asked. Concerning the epistemological dimension: what new forms of knowledge are produced? Concerning the bio-political dimension: how does this affect power relationships? And finally, concerning the ethical dimension: what new practices of the self are allowed to evolve in response to the new knowledge-power constellation?

## The knowledge dimension: the big data of human biology

The emerging Precision Medicine cosmology entails a remapping of illness in large data repositories. Ongoing monitoring and collection of data provides a detailed perspective on illness where inner and outer, body and environment are constantly connected. These new forms of spatialisation and temporalisation gives rise to new knowledge practices.

The PMI Working Group foresees several sources of research data needed to support the scientific opportunities pursued in the cohort: individual demographics and contact information, terms of consent, self-reported measures, behavioural and lifestyle measures, sensor-based observations, clinical data (from electronic health records), baseline health exam, healthcare claims data, research specific observations, bio-specimen derived laboratory data, geospatial and environmental data and lastly ‘other data’, ranging from social networking data to medication purchases (PMI Working Group 2015, pp. 47–48). In the AoU biomedical data are thus procured from various sources; the molecular or microscopic level is represented by the –omics world (genomics, proteomics, metabolomics, lipodomics, transcriptomics, epigenetics, microbiomics, fluxomics, phenomics, etc.), while the macroscopic or ecological world involves epidemiological data of populations and public health informatics (Hampel et al. 2017; Holzinger et al. 2014; Hood and Price 2014). Big Data therefore gains enormous importance because it generates data-driven insights on a systemic level, bringing these different sources together (Holzinger et al. 2014). Big Data science becomes the epistemic base of precision medicine as such.

Big Data analytics generate insights that are “*born from the data*” (Kitchin 2014). Chris Anderson, editor-in-chief of Wired magazine, calls the use of Big Data the end of theory: “Forget taxonomy, ontology, and psychology. Who knows why people do what they do? The point is they do it, and we can track and measure it with unprecedented fidelity. With enough data, the numbers speak for themselves” (Anderson 2008). Big data can be summarized in terms of three major shifts. First of all: Big Data means *N* = all. Secondly, *N* = all means that data can be procured from many different resources. And finally, Big Data implies a shift from *causality* to *correlations* (Sax 2016). Because of these shifts, Big data provides new types of ‘explananda’: it shows the *what* rather than the *why* (Anderson 2008; Mayer-Schonberger and Cukier 2013; Sax 2016). Instead of providing answers to questions, it provides answers, and we should search for the right questions to ask. The main concern is how to translate all these answers into useful knowledge. For the AoU this means that their attempt to gather such a database involves a flood stream of correlation-based information still in need of explanatory power. In many ways it means handing over knowledge-making practices to algorithms and computer intelligence in unprecedented ways.

Kitchin describes several beliefs that underlie big data, such as the idea that big data research doesn’t have to rely on an a priori theory, that data are free of human bias and that, in the case of data, meaning transcends context (Kitchin 2014). Kitchin problematizes these beliefs, arguing that (big) data do not arise from nowhere, and are never free from the “regulating force of philosophy” (Berry 2011, p. 8; Kitchin 2014). He points out that inductive strategies for identifying patterns in data do not occur in a scientific vacuum but are discursively framed, building on previous experiences and established knowledge (Kitchin 2014; Leonelli 2012). This implicit philosophy of big data science obscures former medical cosmologies, those which continue to pass on traditional tools and styles of thinking to the present situation.

Leonelli questions the epistemological ideals presented by Mayer-Schonberger and Cukier in their book *Big Data* (Leonelli 2014). According to Leonelli, an important epistemic weakness of big data is that it tends to be “lossy”. This term, coined by Mayer-Schönberger and Cukier, explains that the sheer quantity of data compensates for certain levels of inexactitude (Mayer-Schonberger and Cukier 2013, p. 200). According to these authors, accuracy becomes less important in view of the volume of the information processed, so that specific forms of incorrectness will be levelled-out. Big numbers do not need to meet the same requirements that are mandatory in traditional research. However, Leonelli argues that the various social, political, economic and technical factors that determine which data are allowed to be processed are non-transparent and difficult to reconstruct by biomedical researchers at the receiving end (Leonelli 2014). With Big Data being ‘lossy’, and the nontransparant factors that determine which data are allowed in, the AoU design might encounter difficulties in trying to live up to the expectations entailed in the label ‘precision’ knowledge. In any event, precision medicine aims to capture the ‘whole domain’ and to collate every quantifiable aspect of an individual’s well-being.

The design of the AoU cohort suggests that the truth about our lives and bodies is mirrored by the data. This involves a new spatialization. Precision medicine creates a new type of space where our views of health and disease become constructed, namely in the database. But precision medicine also entails a new form of temporalisation, because ongoing real-time monitoring and prediction provides constant access to a future situation, resulting in new ways of organising the temporal dimension of disease processes. Deborah Lupton’s argues that through m-health (“mobile health”) technologies, the individual becomes part of a flow of information, a continual loop of production of health-related data. In a similar fashion the object of Precision Medicine becomes a digital cyborg body, through the constant interaction with technology, i.e. monitoring (Haraway 1988; Lupton 2012). Conclusively, framing scientific research in terms of big data will yield correlation-based hypotheses. On the one hand, a big data focus could point to completely new insights that surpass our existing frameworks, but on the other hand relying on databases as such obscures previous traditions and obliterates research subjects by replacing them with mere data (Zwart 2016b).

## Technologies of power; the digital consumer

Foucault in the 1970s showed a growing interest of governments into the health conditions of their populations, leading to the emergence of ‘bio-power’. The power of a nations depends on the health of its citizens (Zwart 2005, p. 36). Disciplines such as statistics or demography are ways to register and control behavior, ways to acquire power over ‘bodies’. In Precision Medicine, medical knowledge is gathered and produced by powerful institutions, shaping and influencing the way of life of individuals. The PMI Working Group suggested that a new era of healthcare is about to flourish and that the AoU can only succeed when individuals become actively engaged in medical practice;

Coincident with advancing the science of medicine is a changing culture of medical practice and medical research that engages individuals as active partners – not just as patients or research subjects. We believe the combination of a highly engaged population and rich biological, health, behavioral, and environmental data will usher in a new and more effective era of American healthcare.’ (PMI working group 2015, 1)

From a philosophical perspective, the idea to engage individuals as active partners raises suspicion. The call for partnership’ poses the question whether there is really a new role for individuals (framed as partners), or whether we should rather see it as a strategy for making citizens responsible for depositing their data in a digital panopticon, as citizen-managers of their everyday life; empowered to make the ‘right’ choices? (Devisch and Vanheule 2015). Patients are increasingly framed as consumers of health technologies, and the medical domain seems to be moving away from governments toward private companies.

The focus on health data in combination with this drive to frame individuals as active partners, has significant consequences for the question *who* will be able to influence the medical’ecosystem. The data sharing community envisioned by the *All of Us* program entails an infrastructure for the assemblage of multiple types of biomedical data which will be managed by a *Data and Research Centre*. Access can be given to researchers, ranging from community colleges up to top healthcare research institutes and industries, but also for citizen scientists, who can propose studies using this information (NIH 2018b). The AoU design requires new forms of collaboration between governmental organizations, research institutions and industry (AoU online-‘partners’) and this is an important element of the institutional base of precision medicine, namely public–private partnerships. Google’s Verily for example (formerly known as Google life sciences) will play a leading role in the data and research centre embedded within the AoU design (Heath n.d.; Lash 2016; NIH 2016, p. new releases; Philippidis 2016). Similarly, Vibrent, a tech company that specialises in AI and machine learning, will fulfil a role as participant technology systems centre (NIH 2018b; Vibrent Health n.d.). These public–private partnerships imply a distributed form of knowledge production where hospitals, research institutes and high-tech entrepreneurs have to work together. (Lupton 2012; Prainsack 2014; Swan 2009). The sharing of information generates new distributions of roles for the stakeholders involved (Aronson and Rehm 2015; Prainsack 2015).

Mayer-Schönberger and Cukier describe an important feature of data called ‘option value’. Rather than being simply consumed, data stay intact and therefore can be accessed and reused over and over again. Databases remain valuable or may even increase in value for addressing future questions. This has been the business model for companies such as 23andme. The company 23andMe acquired PatientLikeMe, a promising social health network, and has been given the role of ‘participant center’ within the AoU design (NIH 2018b). The concern here is to prevent health data from becoming capitalized by private industry (Dickenson 2013). Data can re-inform research questions over and over again and by acquiring intellectual property rights and patenting specific algorithms, private parties may increase their profit. Therefore, access to the AoU database allows private parties to harvest profitable insights.

The rhetoric of patient empowerment is used by powerful commercial actors (Prainsack 2014). When commercial actors mobilize the rhetoric of citizen science and participatory medicine, Prainsack argues, this means alignment of profit orientation and health idealism and this might endanger true empowerment. Similarly, Juengst et al. (2012) speak of “capitalizing” the open-source ethos and urge their readers that they should not assume that patient empowerment always yield positive outcomes for patients. Empowerment may function as an instrument to create demand, or as a device for compliance, or as a way to inflate patients’ responsibilities (Juengst et al. 2012; Prainsack 2014). Other scholars also suggest that the free labor that the customers put in by sharing their health data is at odds with not sharing any financial profits with those who have provided the data, and that patents often erect barriers to research by charging licensing fees (Rimmer 2012).

Although the AoU design might be an attempt to counter this trend and to take the participation aspect seriously, similar risks may nonetheless be involved. Important here is that access does not by definition solve the problem, for gathering large amounts of data is a powerful draw (Leonelli 2014). Until now, the ‘finders keepers’- principle is at work here, criticized by Sax (2016), which enables outsiders to access datasets to dig for ‘gold’, leading to asymmetries and injustices. Any party that discovers some sort of correlation becomes the owner of that information and may patent a particular sequence, for example. In the context of AoU, it remains unclear how profits and benefits will be shared with participating individuals. Individuals are likely to become an exploitable resource, handing off data to external, increasingly powerful parties, while powerful commercial actors are actively involved in the AoU design and in shaping participants’ ‘lifestyle’ choices.

At the same time, however, new opportunities for research have been created which may significantly redistribute power roles for patients. Examples of this democratization of research could also be seen when PatientsLikeMe first started, a social network that relied on quantified self-tracking (Swan 2009). Such networks have proven to make clinical trials more efficient. A PatientsLikeMe patient gathered 250 ALS patients to self-experiment with Lithium (Arnst 2008; Swan 2009). According to Swan, their co-ownership of the health care process and the related issues highlight the possible role individuals could have in participatory medicine.

Devisch et al. argue that if we want to understand the willingness to engage with the medical domain (by sharing health data as in the case of the AoU or via direct-to-consumer genetic testing as in companies such as 23andMe) we should realize that these developments are part of an era of ‘medical consumption’ (Devisch and Van Hoyweghen 2011), so that the neoliberal zeitgeist seems an important vector in the development of precision medicine. (Dickenson 2013; Vogt et al. 2016a, b). In this context we should keep in mind that big data favors people who are more digitally connected (Collins 2015). Thus, articulating the power dimension allows us to decipher a tension between the neoliberal demand to share personal information and the positive ‘right’ to become a member of the AoU community.

## Technologies of the self; biomarker-based health promotion

The AoU program picks up on a trend where medical technologies have become mobile, home-based and consumer-operated, thus enabling remote monitoring. Growing interest in using iPhones, wearables and in-home devices will allow the medical domain to draw near (NIH 2016, p. 8). Individuals are empowered to become self-reflective agents, through the use of smart mobile devices, but at the same time they are more closely monitored than ever by something other than themselves. Individual biomedical signatures foster forms of decision-making that are optimised for your personal health status, while continuous sharing of health data creates a frame of reference for making such decisions. Constant monitoring of health data requires individual to become the managers of his own health, but the ‘norm’ of healthiness is set by an external expert authority (Harvey 2009; Zwart 2007). From an ethical perspective, however, it can be argued that personalised health data (about personal susceptibilities and the risks involved in diets, life-style, career choice, etc.) allow individuals to constitute themselves as responsible subjects, empowered to develop an evidence-based life-style of their own.

In the 1980s, Foucault became interested in the ways individuals manage to transform themselves into moral subjects, using and developing technologies of the self. Whereas precision medicine might lead us to assume that the NIH is hoarding individual data into a digital panopticon, so that healthy behavior can be ‘engineered’, the PMI can also be seen as a space where practices of freedom are allowed to emerge and where individuals can develop a moral lifestyle (Zwart 2005, pp. 37, 39). It enables individuals to address the question what kind of life they want to lead. However this question should not merely pay attention to the ethical dimension as such. Rather we should realize that practices of the self emerge in the folds and margins of these power-regimes, guarded by powerful institutions that shape and influence ‘life’. Thus, new subcultures such as the quantified self-movement emerge in the interspace defined by the three axes. (Sharon and Zandbergen 2016).

While Lupton argues that the web 2.0, the internet of things and the use of wearables have made it possible to directly tailor and target health messages, and that the use of these technologies promote techno-utopian, enhancement and healthist discourses (Lupton 2012, 2013), Sharon’s ethnographic work, studying the Quantified Self Community, offers an entirely different perspective on self-tracking (Sharon and Zandbergen 2016) Members of this community gather data about themselves through self-tracking and share their findings within this community. Sharon’s analysis confirms, however, that, within this community, self-tracking serves other purposes over and above healthism or data fetishism. Self-tracking can, for example, foster practices of mindfulness. Self-tracking and investigation can serve as a practice of resistance against a social norm, or as a communicative and narrative practice: a technology of self-expression (Sharon and Zandbergen 2016). Along this line of thought, precision medicine may indeed provide meaningful new opportunities for probing new practice of the self.

In “The Obliteration of Life”, Zwart (2016b) explored the emergence of technologies of the Self in the “terabyte” age. Human individuality, he argues, is captured in high resolution precision portrayals, but at the same time lost and dissolved in massive data streams. Paradoxically, rather than enhancing self-management, personal -omics portrayals give rise to a molecularised conscience. They serve as indicators of normalcy equipped with increased precision. Increasingly, bio-citizens are regarded as valuable repositories of information Zwart 2016b). Tracking and sharing molecularised self-assessments play an important role in managing our health, thereby created a digital version of a collective Superego.

Conclusively, the individual participant in the AoU has to learn to manoeuvre through a digital landscape composed of multiple datasets projected against different time frames and lifestyles. This context challenges participants to shape what it means to be a member of the AoU community. The question is not whether pariticpants are empowered *or* governed, for the most probable answer is: both. The drive for participation within the AoU can point in both directions.. The subject has to constantly negotiate the meaning of his or her data, and these data can enhance self-expression while at the same time confronting us with a forbidding Superego.

## Conclusion

Following Boenink and others I argued that tools for molecular biology and the development of biobanks, software and algorithms have provided the opportunity for the AoU cohort to flourish. This has lead to a focus on prediction and prevention, providing biomarker-based risk estimates. These developments seem to demarcate the cosmological profile of Precision Medicine. Considering precision Medicine as an emerging cosmology has allowed me to address its possible impact for researchers, practitioners and participating citizens. Additionally, Jewson’s concept enables an assessment of this emerging cosmology so that we may come to terms with several ongoing (practical and conceptual) changes in the biomedical domain, in terms of roles, tasks and responsibilities. Cosmologically speaking, Precision Medicine cosmology combines data-intensive with patient-driven research. Tools, traditions and styles of thinking from former cosmologies are absorbed, but appear in a new constellation and gain new meaning within the AoU design. Foucault’s three axes of inquiry (knowledge, power and self) allowed me to critically assess the Precision Medicine cosmology and show the tensions that arise when Precision Medicine is continued in this direction.

The first tension is an important epistemic paradox. Although the collection of large amounts of health data provides new insights, big data practices at the same time obscure their theoretical and methodological backdrop. Framing scientific research in terms of big data seems to focus on correlation-based hypotheses. On the one hand, a focus on big data could point to completely new insights that surpass our existing frameworks, but on the other hand relying on large-scale databases may obscure previous traditions and reduce research-subjects to mere data.

A second tension concerns the dimension of power, namely the idea that the collection of data is a participatory process, while at the same time it is a strategy to gather data as a tool for health care management. The empowerment-rethoric is a way to create demand, a device for compliance and a way to inflate patient’s responsibilities. In the context of the AoU design (but this applies to Precision Medicine in general), the patient has become a digital consumer. This means that the individual, while being subjected to the neoliberal demand to share personal information, has acquired the positive ‘right’ to become a member of the AoU community.

On the level of self I have argued that Precision Medicine provides a space for self-management by individuals, via meaningful self-examination. Nonetheless, an entrepreneurial logic may actually undermine embodied and experiential knowledge; because individuals are confronted with (or haunted by) expectations and standards derived from big data repositories functioning as a molecular or digital voice of conscience. The subject has to constantly negotiate the meaning of his or her data, which can either enable self-expression, or function as a commanding Superego.

Precision medicine can be considered as an emerging cosmology and the AoU program as an exemplification of this cosmology, allowing us to develop a preliminary diagnostics of what Precision Medicine entails in terms of epistemological, societal and ethical challenges. These considerations may not only deepen our understanding of precision medicine, but may also inform ethical and policy agendas. The next step is to address these issues in the public arena and to initiate deliberations on how to shape and navigate the political ecosystem of precision medicine.

## Footnotes

1 iPOP means Integrative Personalized Omics Profile ans is currently being developed by prof. Snyder famous for the Snyderlab http://snyderlab.stanford.edu/.

2 In Jewsons 1976 paper a footnote mentions that the notion of cosmology bears close resemblance to Althusser’s ‘problematic’, and Kuhn’s ‘paradigm’, additionally parallel ideas are to be found in Bordieu’s ‘intellectual field’, Foucault’s ‘discursive formation’.

3 Jewson and Nettleton speak in terms of patron and patronage as well i.e. the economical organization of the relationship. Because this paper intends to show a shift in focus in the conceptualization of illness, and with that a changing role for medical investigator these concepts are left out.

4 For the sake of coherence I decided to stick to the ‘sick-man’ as Jewson originally put it although referring to a woman, person or subject might be more appropriate.
